# An Integrative Platform of TCM Network Pharmacology and Its Application on a Herbal Formula, *Qing-Luo-Yin*

**DOI:** 10.1155/2013/456747

**Published:** 2013-04-09

**Authors:** Bo Zhang, Xu Wang, Shao Li

**Affiliations:** ^1^Bioinformatics Division and Center for Synthetic and Systems Biology, TNLIST/Department of Automation, Tsinghua University, Room 1-107, FIT Building, Beijing 100084, China; ^2^Joint Computational Center of Drug Discovery, Tianjin International Joint Academy of Biotechnology & Medicine, Tianjin 300457, China

## Abstract

The scientific understanding of traditional Chinese medicine (TCM) has been hindered by the lack of methods that can explore the complex nature and combinatorial rules of herbal formulae. On the assumption that herbal ingredients mainly target a molecular network to adjust the imbalance of human body, here we present a-self-developed TCM network pharmacology platform for discovering herbal formulae in a systematic manner. This platform integrates a set of network-based methods that we established previously to catch the network regulation mechanism and to identify active ingredients as well as synergistic combinations for a given herbal formula. We then provided a case study on an antirheumatoid arthritis (RA) formula, *Qing-Luo-Yin* (QLY), to demonstrate the usability of the platform. We revealed the target network of QLY against RA-related key processes including angiogenesis, inflammatory response, and immune response, based on which we not only predicted active and synergistic ingredients from QLY but also interpreted the combinatorial rule of this formula. These findings are either verified by the literature evidence or have the potential to guide further experiments. Therefore, such a network pharmacology strategy and platform is expected to make the systematical study of herbal formulae achievable and to make the TCM drug discovery predictable.

## 1. Introduction

Traditional Chinese medicine (TCM) is a whole medical system deriving from thousands of years of clinical application that has evolved independently from or parallel to allopathic conventional medicine and has been considered as one of the main items of the complementary or alternative medical system [1, 2]. The treatments of TCM formulate the therapeutic use of herbs using the combinatorial principle of Sovereign-Minister-Assistant-Envoy (*Jun*-*Chen*-*Zuo*-*Shi* in Chinese) on the basis of a patient's syndrome (*ZHENG* in Chinese) and attempt to regain the balance state of life and body functions [3]. However, unlike modern drugs developed by targeting a specific protein, understandings of the molecular basis of traditional herbal formulae are still very limited, posing a serious challenge for the modernization of TCM [4]. With the recent advent of high-throughput technologies, experimental analyses of the active ingredients screening and the mechanisms of action of herbal formulae have become increasingly various. Investigators often examine a herbal formula from different facets by combining chemical or metabolic fingerprint [5, 6], pharmacodynamic and pharmacokinetic technology [7, 8], and genomic, proteomic or metabolomics analyses [9–11]. However, herbal formulae with numerous chemical compounds are too complex to be examined solely by conventional experimental approaches. Moreover, a herbal formula contains hundreds of chemical compounds and its therapeutic effects are mainly produced by complex interactions among ingredients [12]. Current experimental methods are restricted to tap into the deeper well and comprehensively elucidate the molecular mechanisms of TCM. The dearth of modern methods in TCM study and deconvolution of complexity of TCM urgently requires new strategies and appropriate approaches.

As the beginning of TCM network pharmacology, we proposed the possible relationship between TCM and molecular networks in 1999 [13] and established a network-based herbal formulae research framework illustrated by a network-based case study on Cold/Hot herbal formulae and Hot/Cold syndromes in 2007 [14, 15]. Shortly after, the age of “network pharmacology” has clearly begun [16, 17], we believe network pharmacology approaches, focused on examining the network connectivity and dynamics as components of drug targets and designing the optimal therapeutic strategies, can reveal the underlying complex relationships between a herbal formula and the whole body. Thus, we further explored the new subject of TCM network pharmacology by updating the research paradigm from current “one target, one drug” to “network target, multicomponent therapeutics,” which refers to the comprehensive analysis for therapeutic effects of herbal formulae on the basis of the identification of the network target underlying a given disease or TCM syndrome as well as the target network of a given herbal formula [15, 18–20]. To date, accumulating evidence suggests that the network pharmacology analysis is a powerful way to study the molecular mechanisms that are responsible for combinational effects of herbal formula [18, 20–25]. For example, Sun et al. presented a network analysis to explore the mechanism of anti-Alzheimer herbal ingredients by evaluating the distance between the herbal targets and “Alzheimer-related proteins” in the protein interaction network [21]. Wang et al. used a systems biology model integrating oral bioavailability and drug-likeness screening, target identification, and network methods to analyze the synergistic mechanism of four herbs in combined treatment of cardiovascular disease [22].

In this work, to better recognize the active ingredients in herbal formula and uncover the combinational rules of ingredients, we integrate our previous methods into a TCM network pharmacology platform to illustrate network connections between multiple targets of ingredients in herbal formula and multiple genes of a specific disease. The methods we created for TCM network pharmacology in the past years include network-based disease gene prediction, drug target prediction, drug-gene-disease comodule association, herb network analysis, and synergistic drug combination screening [20, 25–31]. The good performance of these methods had been demonstrated in discovery of bioactive compounds and elucidation of action mechanism for herbal formulae [23, 32].

We further apply this integrative platform to unveil the molecular mechanisms of antirheumatoid arthritis (RA) formula named *Qing-Luo-Yin* (Q-L-Y), including four herbs: Ku-Shen (*Sophora flavescens*), Qing-Feng-Teng (*Sinomenium acutum*), Huang-Bai (*Phellodendron chinensis*) and Bi-Xie (*Dioscorea collettii*) [33]. Here, we revealed the target network of QLY against RA-related key processes including angiogenesis, inflammatory response, and immune response and report that the four herbs may produce interactions for enhancing efficiency and reducing toxicity through acting in concert on the target network closely associated with RA. The *Jun* herb, Ku-Shen, treats the main causes of RA, for example, inflammatory response, immune response, and angiogenesis. The *Chen* herb, Qing-Feng-Teng, serves to augment the anti-inflammatory and antiangiogenesis effects of *Jun*. The *Zuo-Shi* herbs, Huang-Bai and Bi-Xie, are used to modulate the therapeutic effects of *Jun-Chen* herbs and to counteract the side effects of Ku-Shen possibly by targeting some off-target genes (i.e., PTGS1). Moreover, we found that the synergism among major ingredients from Ku-Shen and Qing-Feng-Teng may derive from the feedback loop and compensatory mechanisms (i.e., TNF-, IL1B-, and VEGFA induced NF-*κ*B pathways). We also identified several ingredient groups such as Saponins and Alkaloids that act as active components in QLY using the cluster analysis of their target profiles. The above findings are either verified by the literature evidence or have the potential to guide further experiments. Hopefully, our platform is eventually extensible to other herbal formulae, which provides a reliable and practical strategy to identify active herbal ingredients and potential synergistic pairs, to reveal the mechanisms of herbal formulae and to facilitate TCM drug discovery and modernization as well.

## 2. Materials and Methods

### 2.1. Inputs of the Integrative Platform of TCM Network Pharmacology

**Figure 1:**
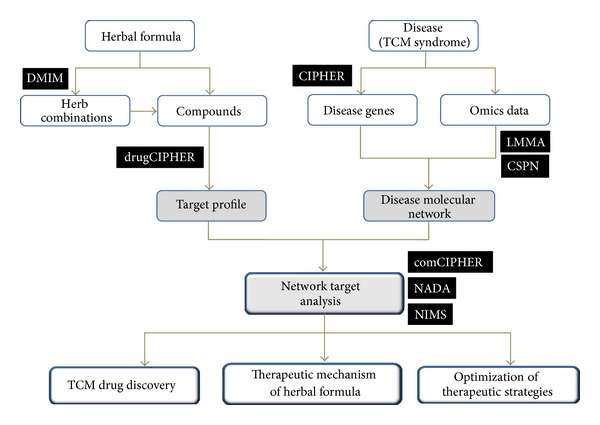
A schematic map of the integrative TCM network pharmacology platform that is based on our network target theory and combined some of our self-developed methods including CIPHER, drugCIPHER, comCIPHER, DMIM, NIMS, NADA, LMMA, and CSPN.

To address the challenges in the study of the molecular basis and combinatorial principle of herbal formulae, we developed a network-based integrative strategy to provide a unified framework as a platform for TCM network pharmacology (Figure 1). This platform contains two different types of entities as inputs: herbal ingredients with known chemical structure, disease-specific genes, and targets of drug treating this disease. Previously, we built a HerbBioMap database to collect the chemical ingredients in 621 herbs [34]. For QLY, we select all available ingredients for each of four herbs in this formula from HerbBioMap and the available literature on the four herbs [34–37]. We also conducted chemical analysis to identify and determine the major ingredients in QLY, for example, Matrine, Kurarinone, Sinomenine, Berberine, and Diosgenin [38, 39]. Finally, after excluding the repeated ingredients, a total of 235 ingredients with 112 in Ku-Shen, 49 in Qing-Feng-Teng, 54 in Huang-Bai, and 20 in Bi-Xie were collected in this study. The structure, canonical name, and CID number of these ingredients were obtained from PubChem [40]. For RA, known RA-related genes were retrieved from the OMIM Morbid Map [41]. Putative RA genes were predicted by our CIPHER method [27]. The known targets of RA drugs were obtained from DrugBank database [42]. For RA disease network construction, these genes and gene products are treated as seeds to obtain their partner genes in the context of the human protein-protein interaction network (HPRD, Release 7) [43].

### 2.2. Predicting Target Profiles for Each Ingredients in QLY

Comprehensively determining compound-target interaction profiles and mapping these on signaling and metabolic pathways will become increasingly necessary for elucidating the mechanisms of action of drugs [44]. *In silico* prediction of target profiles of small molecular compounds especially is a critical step for the study of TCM network pharmacology. Recently, we developed a regression model called drugCIPHER that can predict the links between drugs and target proteins by combining the drug chemical similarity and protein-protein interaction information in a heterogeneous network that correlates chemical, pharmacological, and genomic spaces [26]. In accordance with the rationale of “like attracts like,” this method is based on the hypothesis that drugs with similar chemical structures or therapeutic effects tend to bind to functionally related or modularized target proteins in the molecular network. Thus, drugCIPHER can infer the target profile for a given herbal ingredient with known structure by integrating and making full use of all available FDA-approved drug structures, drug-target interactions, and human protein-protein interactions. The prediction principle of drugCIPHER is also featured in the TCM holism thinking.

In this study, we used the drugCIPHER-CS step in our drugCIPHER method to predict the target profile for each herbal ingredient from QLY. The drugCIPHER-CS score refers to the likelihood of ingredient-target interaction calculated from the correlation between the query ingredient's structure similarity vector in the drug space and the target-related gene's closeness vector in the target space. The resulting proteins with high likelihoods are considered as potential targets of the herbal ingredient. We selected the top 100 proteins with high precision rate as a target profile for each ingredient [26]. We then assembled the target profiles of all available ingredients in every of four herbs and resulted in an integrative target profiles of QLY.

### 2.3. Principal Component Analysis (PCA)

To classify the herbal ingredients from QLY using the predicted target profiles, PCA was performed to reduce the dimensionality of multivariate data into a multidimensional space, allowing for clear visualization of the variation between different herbal ingredients. In this step, we use PCA to reduce the dimension of the target profile of each ingredient in QLY while most of the variance is preserved by linearly transforming the variables into a smaller number, say *n*, of variables that we denote by *z*_1_,…, *z*_*n*_:

(1) $$\begin{array}{c} z_{i}=\sum_{j=1}^{m}\omega_{ij}x_{j},\;\forall i=1,\ldots ,n, \end{array}$$

where *x* = [*x*(1),…,*x*(*m*)]^*T*^ denotes the target profile of each ingredient in QLY and *ω*_*ij*_ is transforming weights with the property of the orthogonality and unit norm:

(2) $$\begin{array}{c} \sum_{j}\omega_{ij}^{2}=1,\;\forall i,\\ \sum_{j}\omega_{ij}\omega_{kj}=0,\;\forall i\neq k. \end{array}$$

### 2.4. Network Target Analysis

To better elucidate the holistic therapeutic effects of QLY, we attempted to figure out the target network and mechanism of action of QLY by our platform. First, genes or proteins involved in RA were compiled by combining RA-causing genes from OMIM and CIPHER prediction [27, 41] and the target proteins of anti-RA drugs from DrugBank [42]. Second, RA-related genes or proteins were used as seeds to fish their partner interacting proteins in the HPRD [43]. The searching of such partner proteins resulted in an expanded network as the RA-specific network. Third, the candidate targets of chemical compounds in each herb predicted by drugCIPHER were mapped into the RA-specific network and were used as a new query to identify the target network of each herb, respectively. Fourth, in order to understand the possible biological functions of each herb, the functional distribution of these target networks was further examined. Finally, the combinational rationale of QLY was interpreted according to the detailed analysis of comodule associations and enriched biological functions.

### 2.5. Biological Function Enrichment Analysis

For biological functional analysis of QLY, we use the functional enrichment tool of the DAVID database to analyze the enriched GO (Gene Ontology) terms for the assembled target-related proteins of QLY with a false discovery rate less than 0.05 by the Fisher exact test [45, 46]. We only selected the GO functional terms with *P* value less than 0.05 after Benjamini's correction.

## 3. Results

### 3.1. A Self-Developed Platform of TCM Network Pharmacology

The concept of “network target” that we proposed [15, 18–20] is the core of the integrative platform of TCM network pharmacology, by which we hypothesize that the relationship between a herbal formula and a disease or TCM syndrome can be transferred into a network context. The key modules in a disease-specific molecular network are considered as the therapeutic target of a given herbal formula. Thus, we can disclose the action mechanisms and the active ingredients as well as their combinations in a herbal formula from the network target viewpoint. This platform fully integrates our developed methods, in which the good performance has been validated, respectively [25–32], for understanding the therapeutic mechanism of herbal formula from the following three aspects (Figure 1).

1. Network target construction: the construction of a disease-specific network as the therapeutic target, such as the prioritization of candidate genes for a given disease (CIPHER) [27], and construction of disease-specific networks in the molecular level (LMMA) [28] or in the pathway-pathway interaction level (CSPN) [29] by combined knowledge and high-throughput omics data.
2. Target prediction and herbal pair extraction: the prediction of target profiles of herbal ingredients (drugCIPHER) [26, 47] as well as the extraction of common herbal pairs from herbal formulae treating specific diseases (DMIM) [25].
3. Comodule analysis based on the network target: such as drug-gene-disease comodule analysis between drug (ingredient) targets from herbal formula and disease-specific network target (comCIPHER) [31] and network target-based computational screening of active ingredients (NADA) [30] and synergistic therapeutic combinations (NIMS) [20, 48].

Different from conventional herbal formulae research strategies that find active ingredients based on a certain disease, our network target strategy [25–32] can lead to more discoveries of active ingredients against various diseases in a network level by capturing each ingredient's target profile in a genome-wide scale. Thus, our platform has two significant characteristics: discovery-oriented and generally applicable, especially in predicting active ingredients, synergistic ingredient combinations as well as active ingredient groups from a given formula, and providing the comprehensive molecular mechanisms of the formula. Here, we examine the *Qing-Luo-Yin* as a case study in the following sections.

### 3.2. Clustering Active Ingredients in QLY Based on the Target Profiles

**Figure 2:**
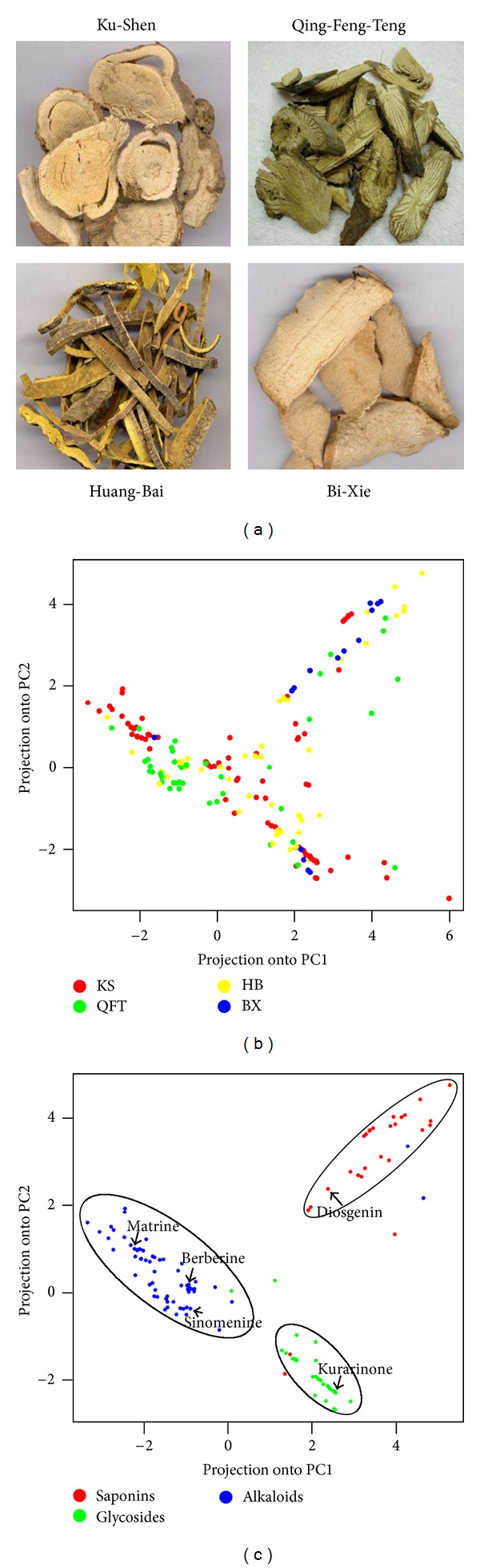
(a) Four herbs Ku-Shen (*Sophora flavescens*), Qing-Feng-Teng (*Sinomenium acutum*), Huang-Bai (*Cortex Phellodendri Chinensis*), and Bi-Xie (*Dioscorea tokoro Makino*) in QLY. (b) and (c) Principal component analysis of the target profiles for each herbal ingredient in QLY. Each dot represents one herbal ingredient plotted against its target profile. Ingredients are color coded according to the four herbs (b) and the three types of the chemicals (c) in QLY. Samples were distributed by their similarity in target profiles using dimensionality reduction. The different chemical types cluster in terms of target profiles can be separated from each other. The four herb clusters in terms of target profiles are partially intermix with each other.

QLY, which derives from a Xin'an medical family [49], is an effective formula in the treatment of arthritis and a typical antiangiogenic herbal formula in TCM [50]. This formula is composed of Ku-Shen (*Sophora flavescens*), Qing-Feng-Teng (*Sinomenium acutum*), Huang-Bai (*Cortex Phellodendri Chinensis*), and Bi-Xie (*Dioscorea tokoro Makino*) (Figure 2(a)) [51]. Our previous studies have revealed the antiangiogenic and anti-inflammatory effects [33] and the network regulation actions of QLY [14].

To further understand the molecular details about how QLY can be administrated on RA, we used our platform to predict the target profiles of each ingredient in QLY and utilized PCA to visually assess the distinction of target profiles of each herb and determine whether herbal ingredients can be grouped. The analysis of dimensionality of all target profiles showed that two first components could account for >60% of the variance present in all targets contained within the target profiles. To see whether the variation retained in the two components contains relevant information about the mechanism of QLY, each ingredient is projected onto the two components as illustrated in Figure 2(b). The PCA analysis showed that four herbs cannot be separated into four independent clusters only according to the target profiles (Figure 2(b)), suggesting that the features of target profiles of the ingredients from different herbs are overlapped. Further, to determine whether the herbal ingredients with the similar chemical properties can be clustered together, the results showed that most herbal ingredients in QLY can be roughly divided into three groups, which exactly were mapped to three types of chemical components, namely, saponins, glycosides, and alkaloids (Figure 2(c)). Figure 2(c) showed that the herbal ingredients in each well-separated cluster may have similar mechanisms of action and can affect different stages or pathological processes of RA in the form of active ingredient groups. Together, these findings indicate that predicted target profiles can be used to identify active ingredient groups leading to similar effects in a herbal formula.

### 3.3. Predicting Active and Synergistic Ingredients from QLY

**Figure 3:**
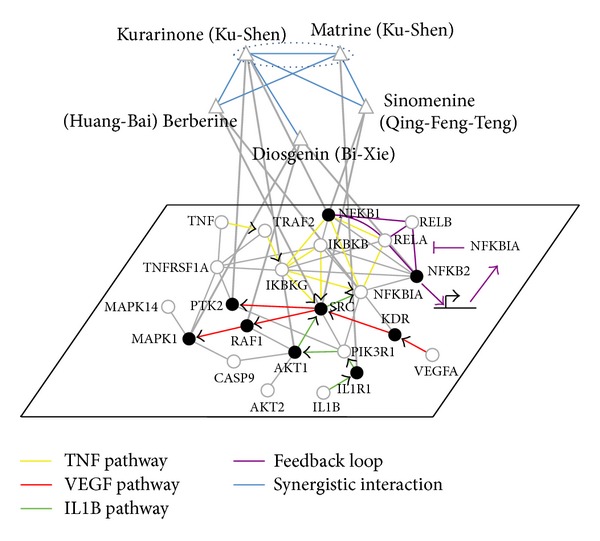
Putative therapeutic mechanism for selected major active ingredients in QLY and potential synergistic pairs. The upper network is the ingredient synergy network (including Kurarinone and Matrine in Ku-Shen, Sinomenine in Qing-Feng-Teng, Berberine in Huang-Bai, and Diosgenin in Bi-Xie), and the lower network is RA-specific molecular network which was constructed manually based on the RA-related pathways and the potential targets of the major ingredients. Herbal ingredients and RA-related genes were represented as triangle and circle, respectively. TNF-, IL1B-, and VEGF-induced pathways were highlighted in color line as indicated. NFKB1, NFKB2, RELA, and RELB can form heterogeneous complex as an important transcription factor in the development of RA. The blue line linking two ingredients indicated synergy with the width of the line correlating with the number of synergistic mechanisms: the wider line represented feedback and compensatory mechanisms and the narrow line represented feedback or compensation.

To examine what ingredients can produce synergistic effects on key pathological processes involving RA, angiogenesis, inflammatory, and immune response and what are the synergistic mechanisms among them, we took advantage of the previous conclusion that synergism may arise from modulations of compensatory actions or feedback loops in the network [20, 52] to estimate the interaction between ingredients. Adapting such criteria, we derived an ingredient-ingredient interaction network in terms of target proteins of each ingredient (Figure 3). We identified six potentially synergistic pairs between main ingredients from Ku-Shen and other herbs, including Matrine and Sinomenine, Matrine and Kurarinone, Matrine and Berberine, Kurarinone and Sinomenine, Kurarinone and Berberine, and Kurarinone and Diosgenin. These prediction results can be supported by the literature evidence. For example, Matrine and Sinomenine were evaluated as a synergistic combination by endothelial cell proliferation assay in previous studies [20] and were confirmed from the mechanism here. As shown in Figure 3, the prediction showed that Matrine can bind to the IL1R1, which suppresses inflammatory and immune response by blocking the NF-*κ*B pathway activated by IL1B. Sinomenine can suppress angiogenesis and inflammation by inhibiting NFKB1 and SRC. Sinomenine may complement Matrine-induced inactivation of NF-*κ*B to reduce its induction of angiogenesis, inflammation, and immune response. In particular, two ingredients from Ku-Shen, Kurarinone and Matrine, may lead to potent synergistic interaction with each other, thus reflecting interactions from different ingredients in the same herb. Because some studies indicated the relationship between NF-*κ*B and oxidative stress in rheumatoid arthritis [53], we inferred that Kurarinone as an antioxidant [54] is likely to inhibit NF-*κ*B activation in terms of predicted target profiles. Kurarinone can inhibit AKT1 and PTK2, which is downstream of IL1B. This may sensitize the effect of Matrine via modulation of NF-*κ*B and AKT1. In addition, four other synergistic pairs were also identified by network-based synergism hypothesis. For example, Matrine and Berberine act on different targets (IL1R1 and KDR) of two cross-talk pathways (IL1B and VEGFA pathway) that regulate the SRC activity. Kurarinone and Sinomenine act on different targets (AKT1 and SRC) of the same pathway (AKT1-SRC-PTK2 pathway) that regulates the NFKB1. Kurarinone and Berberine act on different targets (NFKB1 and KDR) of two related pathways (VEGF and NF-*κ*B pathway) that regulate different targets (SRC and NFKB1). Kurarinone and Diosgenin act on the same type of target (NFKB1 and NFKB2) in the feedback loop (NFKB1-NFKB2-RELA-RELB complex) and different targets (PTK2 and RAF1) of two related pathways.

### 3.4. Target Networks and Combinatorial Rules of QLY

**Figure 4:**
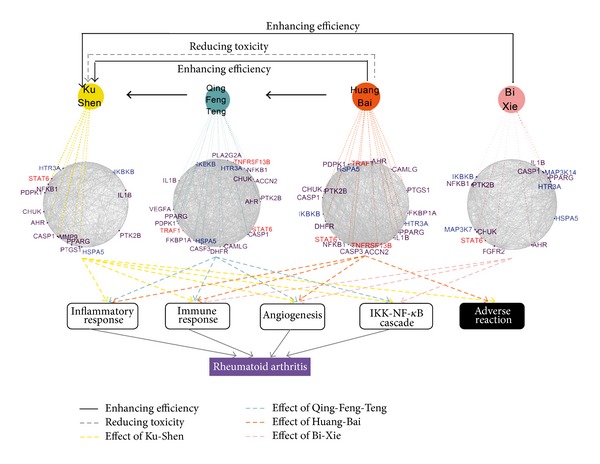
Target network and functional enrichment of the four herbs from QLY. A target network of each herb was uniquely identified by mapping the possible targets of each herb into RA-specific molecular network. The functions for the target networks were obtained by the functional enrichment tool (DAVID). These enriched biological functions are associated with RA. Targeting PTGS1 may lead to some adverse effects. Genes labeled in red color denote RA genes collected from OMIM, genes labeled in purple color denote the targets of the FDA-approved drugs for treating RA, and genes labeled in blue color represent RA genes predicted by CIPHER.

Different from the conventional trial-and-error drug studies, our network-based strategy tries to make the TCM drug discovery predictable and to make the systematical study of combinatorial rules in herbal formulae achievable. As shown in Figure 4, the network target analysis of 235 ingredients in QLY herbs indicated the detailed mechanisms of herb combinations with increased efficacy and decreased toxicity in RA therapy. Ku-Shen, as *Jun* herbs in QLY, acts on the principle RA pathological processes, such as inflammation, immune response, and angiogenesis. Qing-Feng-Teng as a *Chen* herb and Huang-Bai and Bi-Xie as *Zuo-Shi* herbs seem to augment or modulate the therapeutic effects of *Jun* herb through targeting RA-related genes including NFkB1, HTR3A, CASP1, and PPARG. Interestingly, these results of network target analysis demonstrate an unexpected mechanism of QLY for RA therapies, in which Aryl hydrocarbon receptor (AHR) contributing to the pathogenesis of RA [55] is regulated by multiple ingredients in QLY (e.g., Kuraridin, Sophoraflavanone and Xanthohumol in Ku-Shen, Salicylaldehyde and Allantoin in Qing-Feng-Teng, *β*-Elemene in Huang-Bai, and Piperitol in Bi-Xie). Besides, the pharmacological activities of Ku-Shen, Qing-Feng-Teng, and Bi-Xie are associated with targeting the NF-*κ*B pathway. These results demonstrated the synergistic effects among the four herbs of QLY. Our predictions also include the mechanisms of decreased toxicity of *Zuo* and *Shi* herb in QLY. For instance, Xanthohumol in *Jun* herb may cause adverse drug reactions through affecting off-target genes such as PTGS1, resulting in gastrointestinal haemorrhage, haematuria, and abdominal pain [56]. In the target network, we found that cis-limonene oxide phellochinin A and ferulic acid in Huang-Bai may neutralize the adverse effects of Ku-Shen through modulating PTGS1.

In addition, by examining the functional distribution of the potential targets of QLY, we found that the significantly enriched GO terms QLY acted include the key processes in the development of RA, such as inflammatory response, regulation of cytokine production, regulation of angiogenesis, and leukocyte activation (Table 1). Therefore, by modulating these pathological processes, QLY may promote the recovery of network balance from a disease state to a normal state. Together, these results reveal not only the target network of QLY against RA-related angiogenesis, inflammatory response, immune response, and NF-*κ*B activity but also the “*Jun-Chen-Zuo-Shi*” principle of QLY from the connections of functional modules in the network target.

## 4. Discussion

Many common diseases such as cancer and rheumatoid arthritis as well as cardiovascular diseases are complex biological systems caused by multiple molecular abnormalities [57, 58]. During the therapy, many drugs that modulate a single target might not always yield the desirable outcome even if they completely interdict the functions of their direct targets [59, 60]. From a network perspective, the entity that needs to be targeted and modulated must shift from single proteins to entire disease molecular networks [61, 62]. The efficacy of such therapies can be explained by the fact that drugs targeting different proteins in the disease network or pathway could trigger a synergistic response, and their combinations can eliminate compensatory reactions and feedback controls, thereby overcoming the robustness of diseases [63, 64]. These perspectives illuminate that the level of complexity of the proposed therapies should be increased. Interestingly, the properties of TCM herbal formula are consistent with the coming network-based therapeutic strategies. However, currently it is hard to unveil the complex systems embedded in the TCM repertoire, especially the interactions between the complex biological systems of human body and the complex chemical systems of herbal formulae.

To provide a novel route for the systematic studies of herbal formulae, here we report a self-developed integrative platform of TCM network pharmacology (Figure 1) to acquire a better understanding of the underlying mechanisms and combinatorial rules of herbal formula. To the best of our knowledge, this is the first self-developed TCM network pharmacology platform for studying herbal formulae [47, 48, 65]. Indeed, the performance of all methods in the platform have been properly tested, respectively [25–32], and some key methods have been recognized as one of the leading approaches in network biology and network pharmacology [66, 67]. For instance, CIPHER achieves a high-precision accuracy in disease gene prediction that outperforms the state-of-art methods [27], drugCIPHER takes the lead in the genome-wide drug target prediction [26], and NIMS is regarded as a novel method in network pharmacology [67]. These methods can help solve challenging problems in studying chemical and biological basis of herbal formula. For example, drugCIPHER provides a new way to identify target profiles of most ingredients in herbal formulae [26, 47].

In this work, by QLY as a case study, we demonstrate that this platform is effective on identifying bioactive ingredients, synergistic ingredient pairs, and ingredient groups (Figures 2 and 3) and elaborating the combinational rules of QLY (Figure 4), which tentatively validated by statistical approaches as well as literature. For example, the previous experimental studies have shown the anti-inflammatory actions of Matrine, the antiangiogenic effect and anti-IL1B expression of Sinomenine, the immune-regulatory effect of Berberine [68–71], and synergistic effects between Matrine and Sinomenine [20], which proved the outputs of our platform. Although the target networks of QLY need to be further experimentally determined, this platform is useful for uncovering the systematic-level mechanisms that are not easily detectable in experimental studies. For instance, our analysis revealed that not only Ku-Shen and three other herbs may synergize by targeting different biological processes (e.g., angiogenesis, inflammatory, and immune response), but also Huang-Bai may antagonize the adverse reaction of Ku-Shen through some off-target genes (e.g., PTGS1) that deserve further experimental testing.

**Figure 5:**
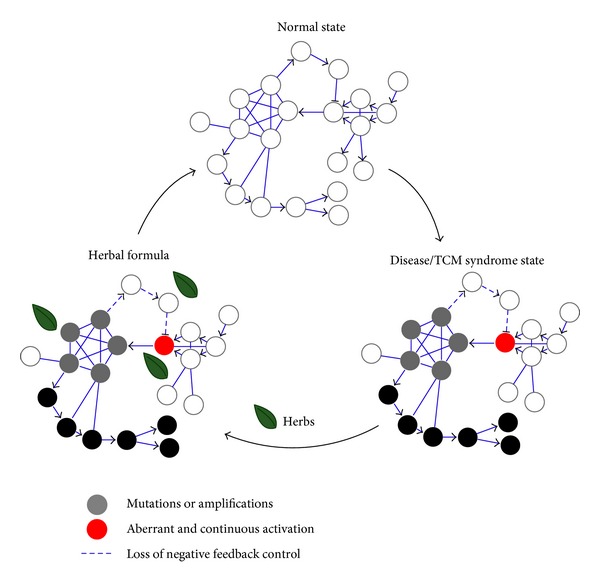
Regulation of network imbalance as an important therapeutic principle of herbal formula. Mutation/amplification and aberrant signal transduction cause the multiple changes of the normal network structure, leading to the imbalance of health state. Combinations of herbs used in herbal formula (substantially certain chemical compounds) can weakly target different proteins within the disease-specific network so as to restore the imbalanced disease state.

The aim of herbal formulae treatment is to adjust an imbalance state of disease-specific network, which refers to the network interaction and node activity or expression in a given disease context deviating from health status (Figure 5). Recent studies of cancer therapy have shown that disease-specific networks are dynamic and can change with time and space in order to adapt to different interventions, resulting in compensatory effects and drug resistance [72, 73]. For a given disease-specific molecular network, the combination of interventions can best restore the disease network to a desired normal state. Thus, our platform can be used to reveal the behavior of network balance regulation featured by herbal formulae. Our results suggest that various ingredients in QLY may weakly target different proteins within the RA molecular network, shut down the whole pathological process by network interaction or biochemical synergism, then maintain a delicate balance of the human body, and finally activate its own capability of disease resistance. In this study, we clarified that the synergistic effects among six main ingredients in QLY are caused by acting on the compensatory pathway and feedback loop in the TNF/IL1B/VEGF-induced NF-*κ*B pathways involved in RA and the synergistic mechanism of QLY is partially associated with the modulation of NF-*κ*B imbalanced network (Figures 3 and 4). Recently, the combination intervention of NF-*κ*B system has provided the evidence for the efficiency of network balance regulation in cancer therapy. For instance, NF-*κ*B-blocking therapies against tumors with constitutive or chemotherapy-induced NF-*κ*B activation represent one of the few examples where inhibition of NF-*κ*B network serves as a homeostatic switch for enhancing genotoxic damage but promotes the secretion of protumorigenic factor, IL-1*β* [74, 75]. In this case, NF-*κ*B inhibition combined with anti-IL-1 therapy will rebalance the adverse effects of perturbed NF-*κ*B network [76]. Indeed, the adjustment of dysregulated NF-*κ*B network implicated in other diseases can help to understand the effects of QLY on RA. QLY is most likely to modulate angiogenesis within inflammation or tumor environment owing to its modulation of the NF-*κ*B network. It is noted that the *Jun-Chen* herb (Ku-Shen and Qing-Feng-Teng) in QLY has shown the therapeutic benefits on tumor development [70, 77]. Recently we also identified and experimentally verified a novel angiogenesis inhibitor, vitexicarpin, from a herb paired with Huang-Bai in QLY [32]. We believe that integrative adjustment of the imbalanced network is expected to be one of the trends of future drug discovery, especially discovery of combinatory drugs from herbal formulae.

In addition, we can also capture the formula-syndrome relationship from a network target viewpoint. The treatment strategy of herbal formula is characterized by guiding the combination of herbs in the light of the imbalance state of the human body, such as TCM Cold and Hot syndromes. We investigated the imbalanced molecular network associated with Cold syndrome and Hot syndrome in the context of the neuroendocrine-immune system and identified several key Hot syndrome-related molecules, such as IL1B, TNF, and VEGF [14]. Our previous results demonstrated that QLY can suppress angiogenesis and inflammation in collagen induced arthritis rats [33]. The present work also demonstrates at the molecular level that QLY as a Cold-natured formula is likely to modulate these network hub molecules of Hot syndrome (IL1B, TNF, and VEGF), aiming to expel the “pathogenetic hot” for curing Hot syndrome-related RA and exert the antiangiogenesis, anti-inflammation, and immune-regulatory actions (Figure 4 and Table 1). All together, these findings evidenced that the mechanism of QLY can be interpreted by its actions on a therapeutic network and its adjustment of the network imbalance state.

As illustrated by *Qing-Luo-Yin*, we demonstrate that the implementation of our TCM network pharmacology platform can not only recover the known knowledge but also provide new findings that deserve further experimental validations for discovering the active ingredients and therapeutic mechanism of herbal formulae. Therefore, this sustainable development platform coupling with the rich experience of TCM is hopeful of shifting the paradigm for conquering complex diseases from the conventional “one target, one drug” to the “network target, multicomponent therapeutics,” offering bright prospects and solid supports for translating TCM from experience-based to evidence-based medicine and accelerating TCM drug discovery as well.

## Figures and Tables

**Table 1:** Enriched RA-related GO terms in the *Qing-Luo-Ying* target network.

Function category	GO term ID	GO terms	*P* value
			(Benjamini's correction)
Angiogenesis	GO:0009611	Response to wounding	1.30*E* − 29
	GO:0045765	Regulation of angiogenesis	1.62*E* − 04
	GO:0010594	Regulation of endothelial cell migration	0.002

Inflammatory response	GO:0006954	Inflammatory response	1.23*E* − 13

Immune response	GO:0001817	Regulation of cytokine production	1.42*E* − 04
	GO:0006955	Immune response	0.002
	GO:0045321	Leukocyte activation	1.17*E* − 05
	GO:0001816	Cytokine production	0.004
	GO:0051249	Regulation of lymphocyte activation	0.001
	GO:0006952	Defense response	2.88*E* − 12
	GO:0042981	Regulation of apoptosis	2.32*E* − 17

NF-*κ*B activity	GO:0051092	Positive regulation of NF-*κ*B transcription factor activity	0.037
